# Transcriptome-Based Synthetic Lethality Predictions to Improve Targeted Therapy/Immunotherapy Treatment Prioritization in Nonmetastatic Breast Cancer: BC-SELECT

**DOI:** 10.1158/2767-9764.CRC-26-0401

**Published:** 2026-10-13

**Authors:** Yewon Kim, Matthew Nagy, Rebecca D. Pollard, Padma Sheila Rajagopal

**Affiliations:** 1 Cancer Data Science Laboratory, Center for Cancer Research, National Cancer Institute, Bethesda, Maryland.; 2 Boston Children’s Hospital, Boston, Massachusetts.; 3 Women’s Malignancies Branch, Center for Cancer Research, National Cancer Institute, Bethesda, Maryland.

## Abstract

Patients with nonmetastatic triple-negative breast cancer (TNBC) or HER2+ breast cancer do not currently have tools to predict treatment response or prioritize options prior to therapy initiation. Pathologic complete response (pCR) is a proxy that requires neoadjuvant treatment. BC-SELECT leverages genetic interactions [e.g., synthetic lethality (SL)/synthetic rescue (SR)] to predict treatment response via pCR from breast cancer gene expression data. BC-SELECT involves (i) “Training: gene-pair identification” that identifies clinically relevant partner genes from large-scale datasets for a given targeted therapy or immunotherapy target and (ii) “Training: parameter tuning” using clinical trials to predict treatment response. We evaluated BC-SELECT’s ability to predict pCR for trastuzumab, poly (ADP-ribose) polymerase (PARP) inhibitors, and immunotherapy, with tuning supported by three trials (*n* = 86) and validation on nine unseen trials (*n* = 722). BC-SELECT significantly predicted pCR in 6 of 9 trials, including all PARP inhibitors [ROC-AUCs, 0.6–0.8; odds ratio (OR), 1.9–3.5] and immunotherapy (ROC-AUCs, 0.7–0.8; OR, 3.2–7.1). BC-SELECT significantly distinguished between pCR and residual disease in 8 of 9 trials. No current standard-of-care, pretreatment approaches predict benefit in nonmetastatic TNBC and HER2+ breast cancer. BC-SELECT leverages genetic interactions to predict treatment response from gene expression data. Our findings support development of BC-SELECT toward studying how to prioritize PARP inhibitors and immunotherapy in nonmetastatic breast cancer.

**Significance:**

BC-SELECT is an algorithm that uses gene expression to predict pCR in breast cancer using targeted therapy and immunotherapy. BC-SELECT predicted response to PARP inhibitors and immunotherapy, distinguished responders and nonresponders, and identified different response patterns among tumors with low *BRCA1/2* gene expression.

## Introduction

Gene expression data have transformed clinical management in nonmetastatic breast cancer. Patients with early-stage hormone receptor (HR)–positive disease undergo testing with tools such as Oncotype or MammaPrint to predict chemotherapy benefit (1–9). HER2+ or triple-negative breast cancer (TNBC), however, lack predictive gene expression tests and require neoadjuvant therapy and surgery to assess pathologic complete response (pCR) to guide adjuvant treatment.

Patients with HER2+ or TNBC may have competing treatment options across neoadjuvant/adjuvant settings, creating a need for predictors. For example, adjuvant PARP inhibitors are approved to treat patients with “high-risk early breast cancer” due to inherited *BRCA1/2* pathogenic variants (most commonly TNBC), and neoadjuvant/adjuvant immunotherapy is approved for “high-risk, early-stage TNBC” (10, 11). Multiple clinical trials in metastatic breast cancer [KEYLYNK-009, MEDIOLA, and JAVELIN (12)] fail to show a clear outcome benefit for combining these therapies. The lack of data in nonmetastatic breast cancer is described explicitly in current National Comprehensive Cancer Network guidelines (13–16). When multiple targeted therapies/immunotherapies are possible, sequential ordering may offer the same benefits without the toxicity of combinations.

Many published computational approaches demonstrate the prognostic and predictive potential of gene expression in nonmetastatic TNBC or HER2+ breast cancer, but these approaches must often navigate practical hurdles toward clinical application, such as feasibility of deployment (as with the use of single-cell sequencing, for example) or lack of retrospective data availability from the clinic (as with the use of only cleaned research datasets; ref. 17). Recent studies support “immune-related” gene signatures to predict immunotherapy response (18–20). HER2 gene expression is evolving alongside immunohistochemistry and FISH (21, 22). Broader transcriptomic data can now be routinely obtained on commercial testing and identifies not only fusions for FDA-approved therapeutic indications but also gene expression levels, positioning it as a real potential biomarker (23, 24).

Synthetic lethality (SL), a form of genetic interaction in which two genes must be concurrently inactivated to cause cell death, is a mechanism of action with known clinical relevance in breast cancer (25, 26). PARP inhibitors are approved to treat breast cancers with inherited *BRCA1/2* pathogenic mutations due to the SL between *BRCA1/2* and *PARP1/2/3* (10, 27, 28). Other genetic interactions, such as synthetic dosage lethality (SDL), in which one gene must be overexpressed and another underexpressed (29), or synthetic rescue (SR), in which inactivation of one gene that would cause cell death is ameliorated by altered activation of another gene, are being actively studied for translational and clinical application (30).

Computational approaches to identify genetic interactions through gene expression have been recently developed. SELECT (31) uses large-scale cell-line and pan-cancer patient tumor transcriptomic data to identify clinically relevant SL/SR partners for cancer therapies. A commercial adaptation, ENLIGHT (32), focuses on advanced cancers. Both algorithms predict treatment response in retrospective clinical trials and demonstrate SR’s ability to predict immunotherapy response (31, 32). However, their testing in nonmetastatic breast cancer was limited. Evaluated breast cancer therapies included only endocrine therapy or trastuzumab without other targeted therapies or immunotherapy.

Accordingly, we present BC-SELECT, designed to facilitate prediction of response to current and future targeted therapy and immunotherapy in nonmetastatic breast cancer. We evaluate BC-SELECT’s ability to predict pCR for trastuzumab, PARP inhibitors, and immunotherapy by tuning on three trials (86 patients) and validating on nine unseen trials (722 patients). BC-SELECT predicts treatment response to PARP inhibitors—not previously shown by SELECT or ENLIGHT—and immunotherapy across breast cancer subtypes, positioning it as a tool to eventually prioritize targeted therapy and immunotherapy in nonmetastatic breast cancer.

## Materials and Methods

All datasets used in this article are publicly available. Clinical trials and data collection were reported to be in accordance with the Declaration of Helsinki and National Institutes of Health (NIH) Institutional Review Board (IRB) guidelines. Aggregate de-identified data were used, and the use of these data was determined not to require individual patient informed consent per the NIH IRB.

BC-SELECT was tuned and evaluated using publicly available neoadjuvant breast cancer clinical trial datasets with transcriptomic profiles and corresponding pCR outcomes. Only pretreatment primary breast tumor biopsy samples collected before initiation of neoadjuvant therapy were used. pCR was determined by each trial after completion of therapy using surgical specimens. Independent breast cancer cohorts [The Cancer Genome Atlas (TCGA; ref. 33), Molecular Taxonomy of Breast Cancer International Consortium (METABRIC; ref. 34), and Sweden Cancerome Analysis Network - Breast (SCAN-B; ref. 35)], which do not include both gene expression data and individual-level pCR data, were exclusively used for survival-based gene-pair identification filtering. Details of tissue collection as reported by each study are provided in Supplementary Table S1.

### Gene-pair identification

In BC-SELECT, we first identify pairs of genes that interact via SL, SDL, or SR, in which one of the genes was a drug target. For targeted therapies, we test for SL/SDL gene pairs. DrugBank serves as the reference for drug target genes (36). For immunotherapy, we test for SR pairs. *PDCD1* and *CD274* were our drug targets. We focus on targeting PD-1/PD-L1 given the availability of appropriate trial data. Pathway analysis of partner genes was performed using the Gene Ontology (molecular function) database in PANTHER v.19.0, with a multiplicity-adjusted *P* value of 0.05.

**Figure 1.**
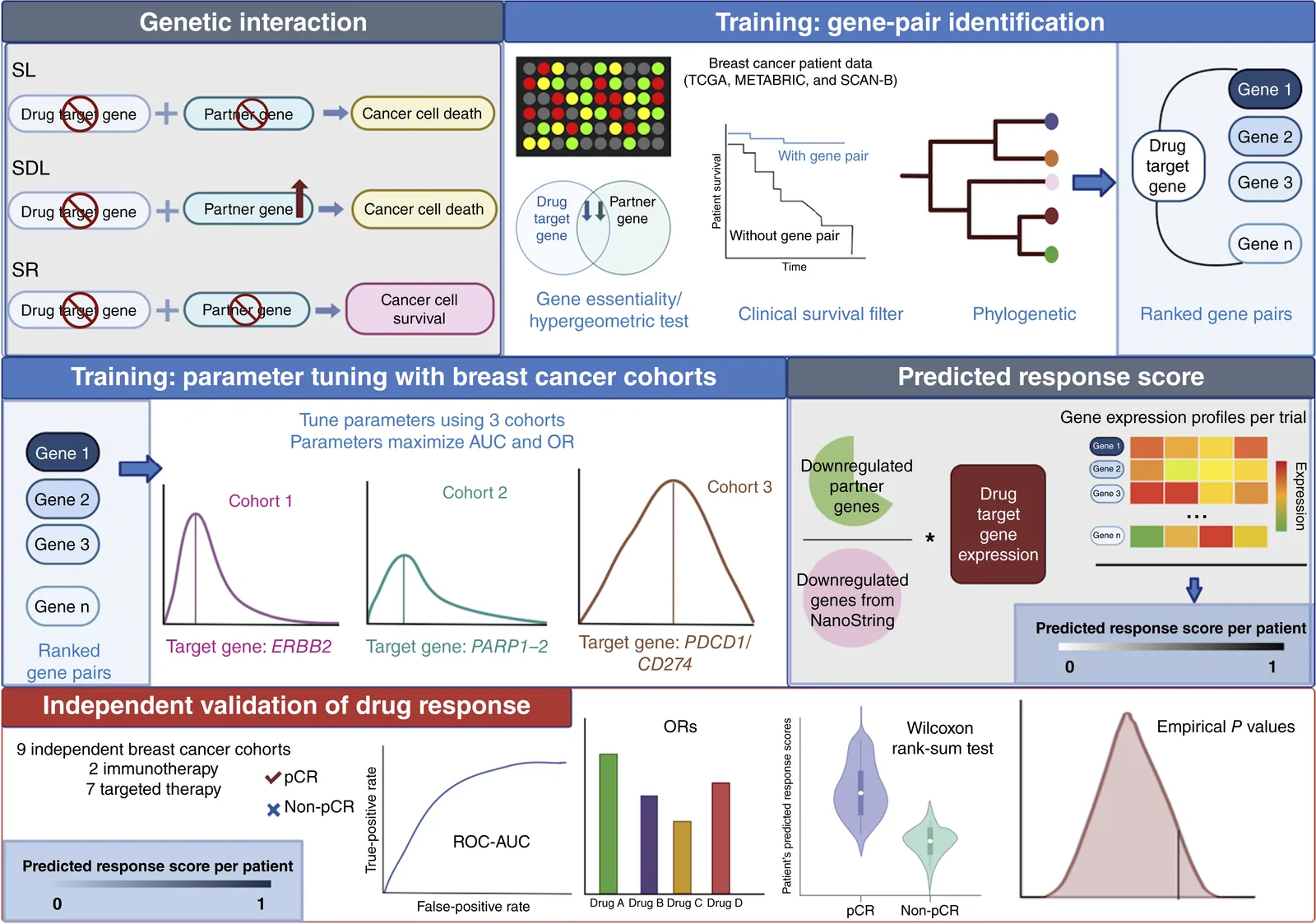
Graphical overview illustrating the adaptation of the BC-SELECT pipeline. BC-SELECT training involves two steps (in blue): gene-pair identification and parameter tuning. The first step focuses on identifying gene pairs involved in genetic interactions. We then adjust parameters to optimize drug response prediction performance using a subset of available clinical trials during the second step. We then validate this in unseen clinical trials and in nine additional validation breast cancer clinical trials (red), with explanations (gray).

Gene pairs must pass the following three filtering steps in BC-SELECT:

(1) *Large-scale gene-pair screen with in vitro tumor cell-line data and in vivo tumor data*. For targeted therapy, we perform a Wilcoxon rank-sum test to identify pairs of genes from published studies on genetic dependencies/SL screening, from databases such as DepMap and the Cancer Cell Line Encyclopedia (37–42), and published studies on genetic relationships to pharmacology (43–45). For immunotherapy, we perform a hypergeometric test (46) to identify pairs of genes observed as underexpressed more frequently than expected by chance in three publicly available datasets of gene expression data [either microarray or normalized RNA sequencing (RNA-seq)] from patients with breast cancer: TCGA (*n* = 1,075; ref. 33), METABRIC (*n* = 1,989; ref. 34), and SCAN-B (*n* = 2,969; ref. 35). Gene underexpression was defined as the lowest relative tertile of expression within a dataset, and gene overexpression was defined as the highest tertile. Each dataset is separately assessed to avoid batch effect. Significant gene pairs are advanced to Step 2, in which statistical significance is determined using a prespecified false discovery rate (FDR)–corrected threshold of *P* = 0.2 to avoid overly conservative exclusion of potentially relevant genetic interactions.
(2) *Cox proportional hazards models for patient survival* that include the genetic interaction pairs identified in Step 1. Clinical variables are included as covariates in addition to gene expression values to determine if gene expression remains significantly associated with patient survival in a clinical context. Although our outcome of interest as discussed in this article is pCR and we use this in the parameter tuning and validation steps, many datasets do not include individual-level treatment response with transcriptomic data. Accordingly, we use survival data in the gene-pair identification step. This serves as a filter for any gene-pair interactions with potential clinical relevance. To incorporate gene expression into a Cox proportional hazards model, we split expression for a gene into tertiles (upregulated in the top tertile, “no change” in the middle tertile, and downregulated in the bottom tertile). We incorporate patient-specific covariates, including age, stage, sex, and race, as these covariates are available in each dataset.
 For targeted therapy: We perform six separate Cox proportional hazards analyses: one for SL gene pairs and one for SDL gene pairs, each performed separately in TCGA (33), METABRIC (34), and SCAN-B (35) datasets to avoid batch effect confounding.
 For immunotherapy: We perform three separate Cox proportional hazards analyses using TCGA (33), METABRIC (34), and SCAN-B (35) datasets and SR gene pairs.
 Significance of a given candidate gene pair within at least one Cox proportional hazards model is determined by using the multiplicity-corrected marginal *P* value of covariates for gene expression and the *P* value from the associated log-likelihood ratio test. If a gene pair has multiple significant *P* values, the minimum *P* value across these analyses is taken as the representative value for that gene. Again, significance is determined by a prespecified FDR-corrected threshold of *P* = 0.2 to avoid overly conservative gene-pair exclusion. Gene pairs significantly associated with survival through at least one Cox proportional hazards analysis are advanced to Step 3.
(3) *Evolutionary analysis of preserved gene expression relationships* using the Euclidean distance of phylogenetic similarity scores across multiple species (47) and feature weight (48).
 For immunotherapy: We compute the phylogenetic similarity score between both *PDCD1* and *CD274* and an associated candidate partner gene. The final phylogenetic score for the partner gene is determined as the smaller value between the two similarity scores generated for *PDCD1* and *CD274*.
 We identify phylogenetically linked candidate partner genes using a threshold (acceptable maximum Euclidean distance between two linked genes) of 10.5 for targeted therapy (46) and 23.15 for immunotherapy (31). Additional details are provided in Fig. 1 and Supplementary Methods.

### Parameter tuning

We use three breast cancer clinical trials with individual-level response data for parameter tuning: GSE66399 (49), GSE160568 (50), and NCT02489448 (Supplementary Table S1; ref. 51). This approach avoids overfitting issues while achieving flexibility across trials. We performed a grid search to optimize the following parameters: (i) the number of significant gene pairs required to predict treatment response (ranging 10–100) and (ii) the odds ratio (OR) threshold to distinguish pCR versus non-pCR.

For targeted therapy, we select the top 55 SL/SDL gene pairs, with ranking based on adjusted significance from Cox proportional hazards. We used only either SL or SDL depending on predictive performance to avoid mixed direction of effect. For *immunotherapy*, we selected the top 10 SR gene pairs, with ranking based on phylogenetic similarity scores. We used an OR threshold of greater than 0.51 for “pCR.”

Note: We include the “parameter tuning” step to optimize the parameters of significant gene pairs and OR threshold across the tested therapies. Although this step improved validity, it is not strictly required. The gene pairs fed into “parameter tuning” from the “gene-pair identification” step are already derived from large-scale datasets with intensive filtering.

### BC-SELECT predicted response score

The predicted response score in BC-SELECT was the product of the partner gene expression ratio and the drug target gene expression level. Calculation for each element is described in detail in the Supplementary Methods.

The breast cancer–specific and immune-specific gene lists used as part of the partner gene expression ratio calculation were selected through commercial NanoString panels (52, 53). The two NanoString gene lists are provided in Supplementary Table S3.

### Validation trials

We used publicly available phase II to III clinical trials with at least 25 patients with stage I to III breast cancer, individual-level gene expression data from the primary tumor, and reported treatment response. We selected pCR to measure response, as this treatment response variable could be harmonized across clinical trials. We used pCR as reported by the clinical trials—a binary measure of either 0 (residual disease/non-pCR, including Residual Cancer Burden levels) or 1 (complete response). All trials used the definition of pCR as the absence of residual invasive tumor in the breast and axillary lymph nodes (54). Treatment response for these trials served solely as the “ground-truth” comparator versus BC-SELECT predicted response score.

We included a total of nine clinical trials, with seven across three targeted therapies (trastuzumab, olaparib, and veliparib; refs. 20, 55–60) and two clinical trials for immunotherapy (Supplementary Table S1; refs. 20, 60). Where trials recruited HR+ patients, evaluation of these patients was handled in the same manner as for the patients with TNBC and HER2+ breast cancer.

### Validation of treatment response prediction

We compared BC-SELECT predicted response scores per patient with the actual responses reported in each trial. We calculated ORs with 95% confidence intervals (CI) and *P* values using the Fisher exact test and ROC-AUC and precision–recall curves to assess prediction performance.

Note: GSE173839 (60) involved treatment with two drugs in our study (olaparib and durvalumab). We independently evaluated the predicted response of olaparib and durvalumab and generated two separate BC-SELECT scores, one for each drug, and two separate AUCs.

### Cross-therapy score association analysis

To determine whether BC-SELECT identifies similar or distinct patient populations for immunotherapy and PARP inhibition, we examined the relationship between immunotherapy-related SR scores and veliparib-related SL/SDL scores. Because germline *BRCA1/2* mutation status was unavailable for our datasets, we used *BRCA1* and *BRCA2* expression as a proxy for BRCA deficiency. Samples were ranked by the average expression of *BRCA1* and *BRCA2* in each cohort, and the bottom 15% of samples were selected for analysis. For the immunotherapy-treated GSE194040 (20) pembrolizumab cohort, veliparib-related SL scores were calculated using the previously identified veliparib SL partner genes. Conversely, for the PARP inhibitor–treated GSE194040 (20) veliparib and GSE164458 (59) veliparib cohorts, immunotherapy-related SR scores were calculated using the top 10 SR partner genes identified for immunotherapy analysis. Associations between the two scores were evaluated using Spearman rank correlation, given the modest sample sizes and the possibility of nonlinear relationships between the scores.

### Statistical analysis

For targeted therapy, we perform a Wilcoxon rank-sum test to rank and select gene pairs. For immunotherapy, we perform a hypergeometric test to identify pairs of genes observed as underexpressed more than expected by chance in breast cancer datasets. We use a Cox proportional hazards model as part of the gene pair filtering.

We estimated the statistical significance of BC-SELECT’ predicted response score and components compared with chance using Monte Carlo simulations of the ROC-AUC (Supplementary Fig. S2; Supplementary Methods).

All analyses were conducted using R v.4.2 (Table 1). To control the FDR, we apply the Benjamini–Hochberg (BH) procedure (BH step-up procedure) as a multiplicity correction.

**Table 1.** Statistical methods used for training and validation in BC-SELECT.

​	Statistical method	Section/figure and table
Training: gene-pair identification	Wilcoxon rank-sum test: essentiality, drug screen (Step 1) Hypergeometric test: SR partner for immunotherapy module (Step 1) Cox proportional hazards, likelihood ratio test: survival filtering (Step 2)	Materials and Methods
Validation of drug response: independent data	ORs, Fisher exact test *P* value, and 95% CIs	Figure 2 and Table 2
Validation of drug response: independent data	Wilcoxon rank-sum test	Figure 3
Validation of drug response: independent data	Monte Carlo empirical *P* values	Table 3 and Supplementary Fig. S2
Validation of drug response: independent data	ROC-AUC and precision–recall curves	Table 3, Fig. 4, and Supplementary Fig. S1

To facilitate clarity about the statistical methods, we provide a brief overview of the specific methods used and where these are described in further detail.

#### Key resource identifiers

The resources used were DrugBank (RRID: SCR_002700), Gene Ontology (RRID: SCR_002811), DepMap (RRID: SCR_017655), Cancer Cell Line Encyclopedia (RRID: SCR_013836), TCGA (RRID: SCR_003193), METABRIC (RRID: SCR_028351), Gene Expression Omnibus (RRID: SCR_005012), and R statistical software (RRID: SCR_001905).

## Results

### Overview of the BC-SELECT framework and adaptations to predict pCR for therapeutic options in nonmetastatic breast cancer

Figure 1 shows the BC-SELECT pipeline. BC-SELECT has two parts: “Training: gene-pair identification” and “Training: parameter tuning.” We used large-scale cell-line and breast cancer datasets to find clinically relevant, interacting gene pairs A and B [in which gene A is the target(s) for targeted therapy or immunotherapy and multiple partner B genes are identified]. Expression levels of partner B genes were used to generate a patient’s predicted response score to a treatment targeting gene A. We tuned parameters using one clinical trial that reported pCR per therapy (Supplementary Table S1; Supplementary Methods). We validated BC-SELECT using nine unseen, independent clinical trials in patients with stage I to III breast cancer: four using trastuzumab in HER2+ disease, three using PARP inhibitors in TNBC, and two using PD-1/PD-L1 immunotherapy in TNBC (Supplementary Table S1). Three trials also recruited HR+ patients. Patient responses were evaluated only within their own clinical trials to avoid batch effect or inaccurate cross-dataset normalization.

### Partner gene biology

Partner genes identified by BC-SELECT for therapeutic targets (HER2, PARP1/2, and PD-1/PD-L1; Supplementary Table S2) are associated with complementary pathways. Significant partner pathways (*P* < 0.01) for HER2 SL included microtubule binding (*n* = 9 genes) and tubulin binding (*n* = 9). Significant partner pathways for PARP SL included ATP hydrolysis activity (*n* = 7) and helicase activity (*n* = 5). Partner pathways for PD-1/PD-L1 SDL were most frequently related to MHC protein complex binding (*n* = 2, *CD4* and *CD8A*), although not significant after multiplicity correction. Specific genes are highlighted in Supplementary Table S2. *FLT3*, *PLOD1*, *TRIB2*, and *TK1* overlapped between SDL/trastuzumab partners and SL/SDL/PARP inhibitor partners. No partner genes were shared between targeted therapy and immunotherapy.

### BC-SELECT significantly predicted pCR in response to PARP inhibitors and immunotherapy

Across all trials, complete responders (pCR = 1) demonstrated consistently higher predicted scores from BC-SELECT compared with those with residual disease/non-pCR (pCR = 0).

**Figure 2.**
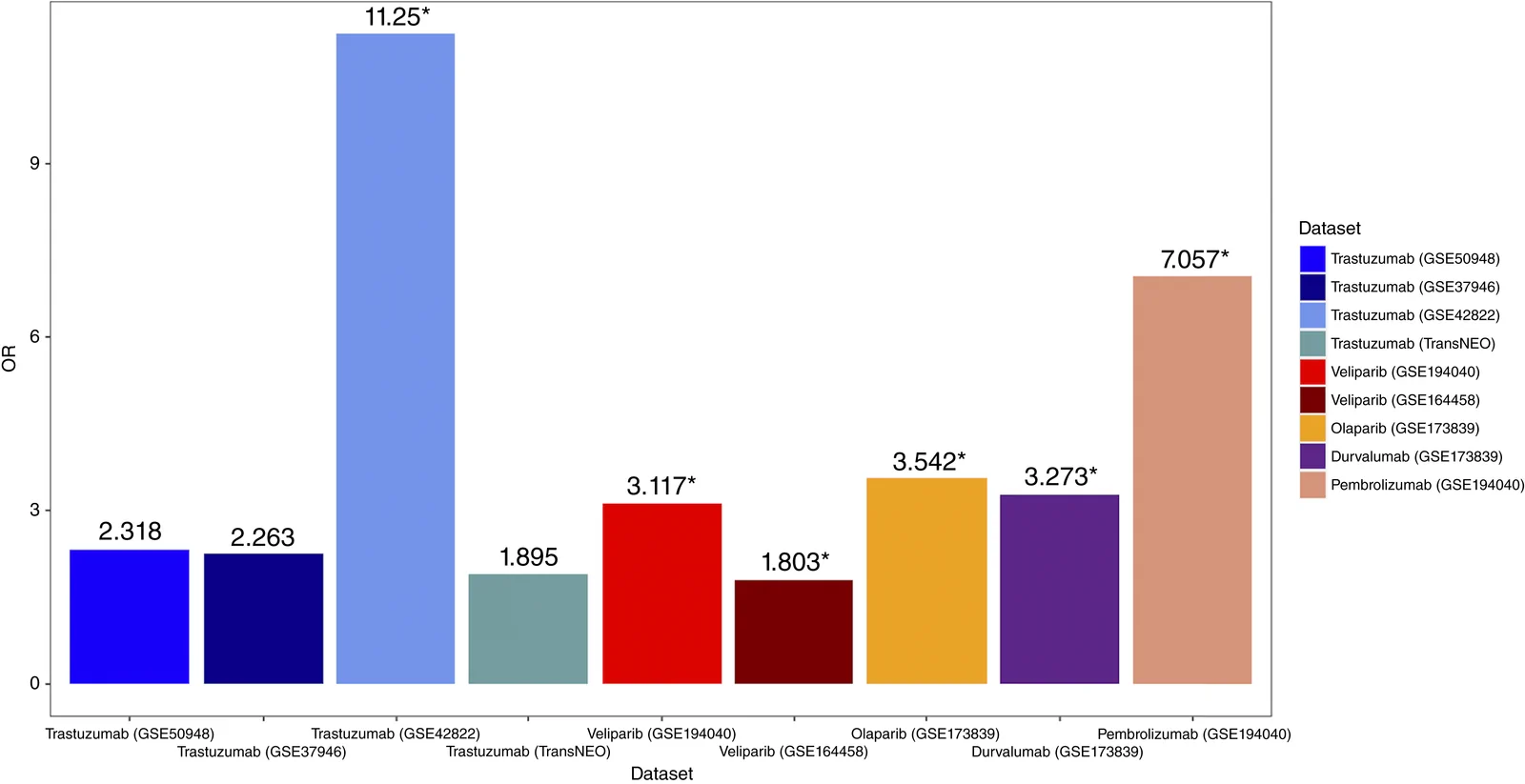
ORs between pCR and non-pCR outcomes using final predicted response scores from BC-SELECT. *X*-axis shows the specific clinical trial, and *Y*-axis shows the OR. Using a threshold of 0.51, we calculated the ORs between pCR and non-pCR outcomes across nine breast cancer clinical trials. Blue/green, trastuzumab; red/orange, PARP inhibitors; purple/pink, immunotherapy. All ORs were greater than 1, and prediction in six trials showed statistical significance (marked with *), with the 95% CI not reaching 1. Detailed 95% CIs of ORs and multiplicity-corrected *P* values from Fisher exact tests are provided in Table 2.

**Figure 3.**
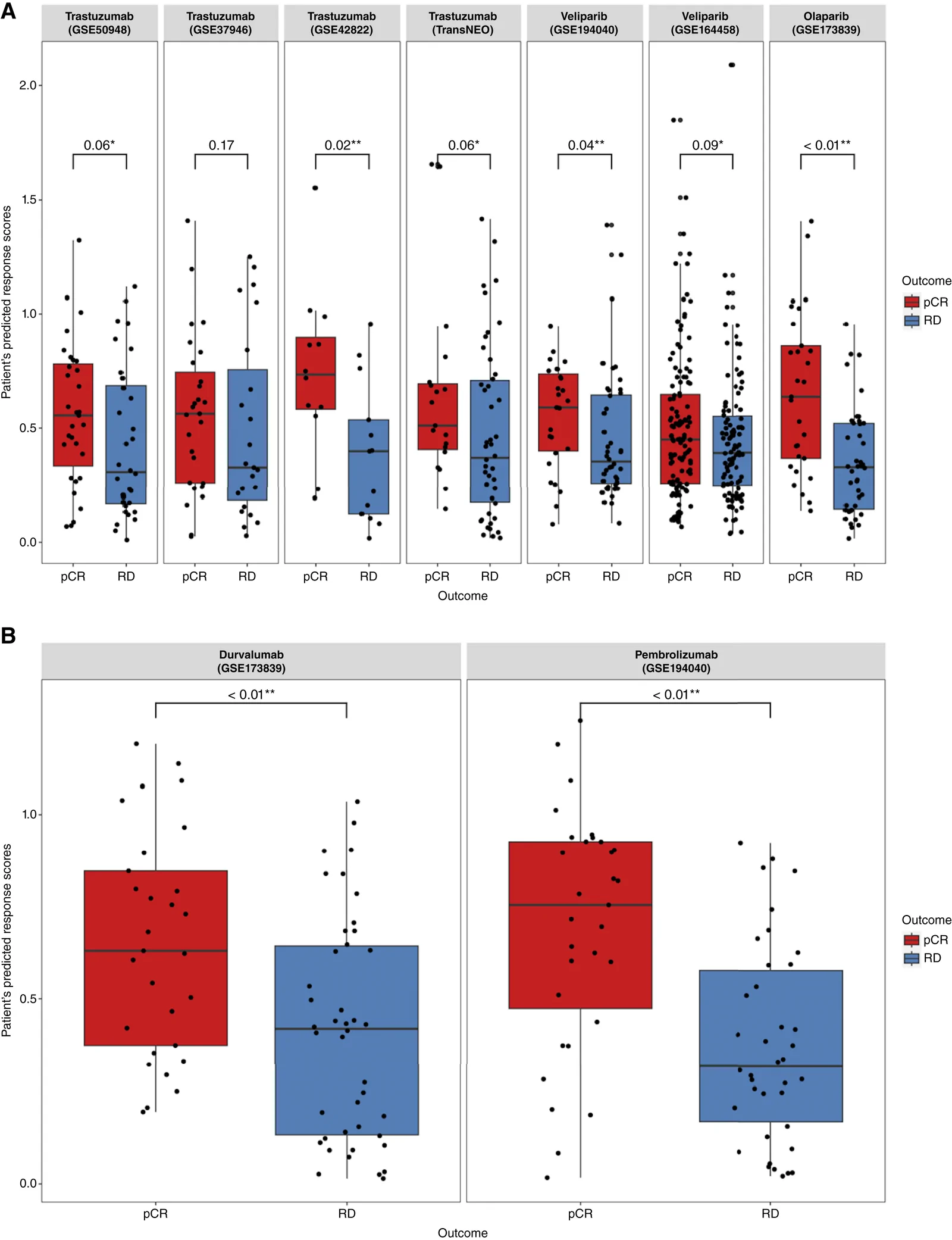
Patient’s final BC-SELECT predicted response scores for pCR vs. non-pCR outcome. Red, pCR; blue = residual disease/non-pCR outcome (RD). **A,** Targeted therapies. *X*-axis shows the specific clinical trial, and *Y*-axis shows the distribution of predicted response scores within the trial. Final predicted BC-SELECT scores were significantly higher in pCR compared with RD in six of seven trials. **B,** Immunotherapy. Final BC-SELECT predicted scores are also significantly higher in pCR vs. RD in both immunotherapy trials. Significance was determined via the Wilcoxon rank-sum test with correction for multiple hypothesis testing. Adjusted *P* values are displayed in each box plot, *, *P* < 0.1; **, *P* < 0.05.

We derived ORs between pCR and non-pCR based on BC-SELECT final predicted response scores (Fig. 2). ORs were statistically significant in 6 of 9 trials using a threshold of 0.51, including all PARP inhibitor and all immunotherapy trials (Table 2). We observed a statistically significant difference between final predicted response scores with pCR compared with non-pCR for both targeted therapy (6/7 trials; Fig. 3A) and immunotherapy (2/2 trials; Fig. 3B; FDR-corrected Wilcoxon rank-sum test).

**Table 2.** ORs between pCR and non-pCR outcome using patients’ final predicted response scores from BC-SELECT.

Treatment category	Drug	Target	Trial	Genetic interaction	OR	95% CI	Fisher exact test adjusted *P* value
Immunotherapy	Durvalumab	*PDCD1* and *CD274*	GSE173839 (60) pCR: 29; RD: 42	SR	**3.273**	**(1.22–8.782)**	0.03**
	Pembrolizumab		GSE194040 (20) pCR: 31; RD: 38	SR	**7.057**	**(2.428–20.514)**	<0.01**
Targeted therapy	Veliparib	*PARP1* and *PARP2*	GSE194040 (20) pCR: 27; RD: 44	SDL	**3.117**	**(1.151–8.438)**	0.07*
			GSE164458 (59) pCR: 127; RD: 110	SL	**1.803**	**(1.049–3.098)**	0.07*
	Olaparib	*PARP1, PARP2,* and *PARP3*	GSE173839 (60) pCR: 29; RD: 42	SL	**3.542**	**(1.307–9.6)**	0.05**
	Trastuzumab	*ERBB2* (HER2)	GSE50948 (55) pCR: 31; RD: 32	SDL	2.318	(0.839–6.404)	0.18
			GSE37946 (56) pCR: 27; RD: 23	SDL	2.263	(0.727–7.047)	0.28
			GSE42822 (57) pCR: 12; RD: 13	SDL	**11.25**	**(1.647–76.852)**	0.05**
			TransNEO (58) pCR: 19; RD: 46	SL	1.895	(0.643–5.589)	0.28

NOTE: All ORs were greater than 1, and six trials showed statistical significance (bold) based on 95% CIs. Adjusted *P* values as determined using the Fisher exact test and multiple hypothesis correction are displayed in the last column: *, *P* < 0.1; **, *P* ≤ 0.05.

Abbreviation: RD, residual disease/non-pCR outcome.

BC-SELECT’s final predicted response score outperformed its two components: target gene expression (gene A) and partner gene expression ratio (genes B; Table 3). For targeted therapy, BC-SELECT’s final predicted response scores predicted pCR significantly better than chance (one-tailed empirical *P* value < 0.025) in four trials (2/3 trials with a PARP inhibitor and 2/4 trastuzumab trials), with AUCs ranging from 0.63 to 0.8, whereas partner gene expression ratio and target gene expression were each statistically significant in two trials. For immunotherapy, BC-SELECT and partner gene expression ratio were both statistically significant for both trials in predicting response compared with chance, but ROC-AUCs were higher with BC-SELECT compared with the partner gene expression ratio (0.71, 0.78 vs. 0.62, 0.66; Table 3). Target gene expression was significant in one of two trials. Precision–recall curves are shown in Supplementary Fig. S1.

**Table 3.** ROC-AUC values and empirical *P* values for BC-SELECT and its score components (partner gene expression ratio and target gene expression) in predicting nine breast cancer clinical trials.

Treatment category	Drug	Target	Trial	Genetic interaction	BC-SELECT ROC-AUC (empirical *P* value)	Partner gene expression ratio, ROC-AUC (empirical *P* value)	Target gene expression, ROC-AUC (empirical *P* value)
Immunotherapy	Durvalumab	*PDCD1* and *CD274*	GSE173839 (60) pCR: 29; RD: 42	SR	**0.71** (0.01)	**0.62** (0.01)	0.70 (0.03)
	Pembrolizumab		GSE194040 (20) pCR: 31; RD: 38	SR	**0.78** (<0.01)	**0.66** (<0.01)	**0.75** (<0.01)
Targeted therapy	Veliparib	*PARP1* and *PARP2*	GSE194040 (20) pCR: 27; RD: 44	SDL	**0.66** (0.02)	**0.59** (0.02)	0.63 (0.1)
			GSE164458 (59) pCR: 127; RD: 110	SL	0.56 (0.32)	0.45 (0.99)	**0.61** (0.02)
	Olaparib	*PARP1*, *PARP2*, and *PARP3*	GSE173839 (60) pCR: 29; RD: 42	SL	**0.77** (<0.01)	**0.8** (<0.01)	0.56 (0.27)
	Trastuzumab	*ERBB2* (HER2)	GSE50948 (55) pCR: 31; RD: 32	SDL	**0.63** (0.02)	0.6 (0.05)	0.6 (0.06)
			GSE37946 (56) pCR: 27; RD: 23	SDL	0.59 (0.08)	0.48 (0.25)	0.6 (0.09)
			GSE42822 (57) pCR: 12; RD: 13	SDL	**0.8** (0.01)	0.64 (0.38)	**0.78** (0.01)
			TransNEO (58) pCR: 19; RD: 46	SL	0.64 (0.19)	0.51 (0.79)	0.69 (0.04)

NOTE: We report ROC-AUCs and empirical *P* values, which describe the performance within a Monte Carlo–generated null distribution. ROC-AUC values are highlighted in bold if the ROC-AUC values fall within the top 2.5% of null AUC distributions. For targeted therapies, the reported ROC-AUC value is higher, which was generated using either SL or SDL.

Abbreviation: RD, residual disease/non-pCR outcome.

To confirm that BC-SELECT scores were not inadvertently increased because of an unmeasured retrospective confounder among patients who had received targeted therapy and had better outcomes, as a form of negative control, we compared the theoretical AUC of PARP inhibitor response for both placebo arms in BrighTNess (GSE164458; B and C, 245 patients; SL: 0.51; ref. 59) with the actual AUC (arm A, SL: 0:56). In other words, these participants did not receive a PARP inhibitor, and we sought to assess how BC-SELECT predicts a comparable group of patients with breast cancer that would hypothetically respond to a PARP inhibitor. Similar values suggest no inadvertent inflation.

### BC-SELECT predicted immunotherapy response better than SELECT and ENLIGHT

**Figure 4.**
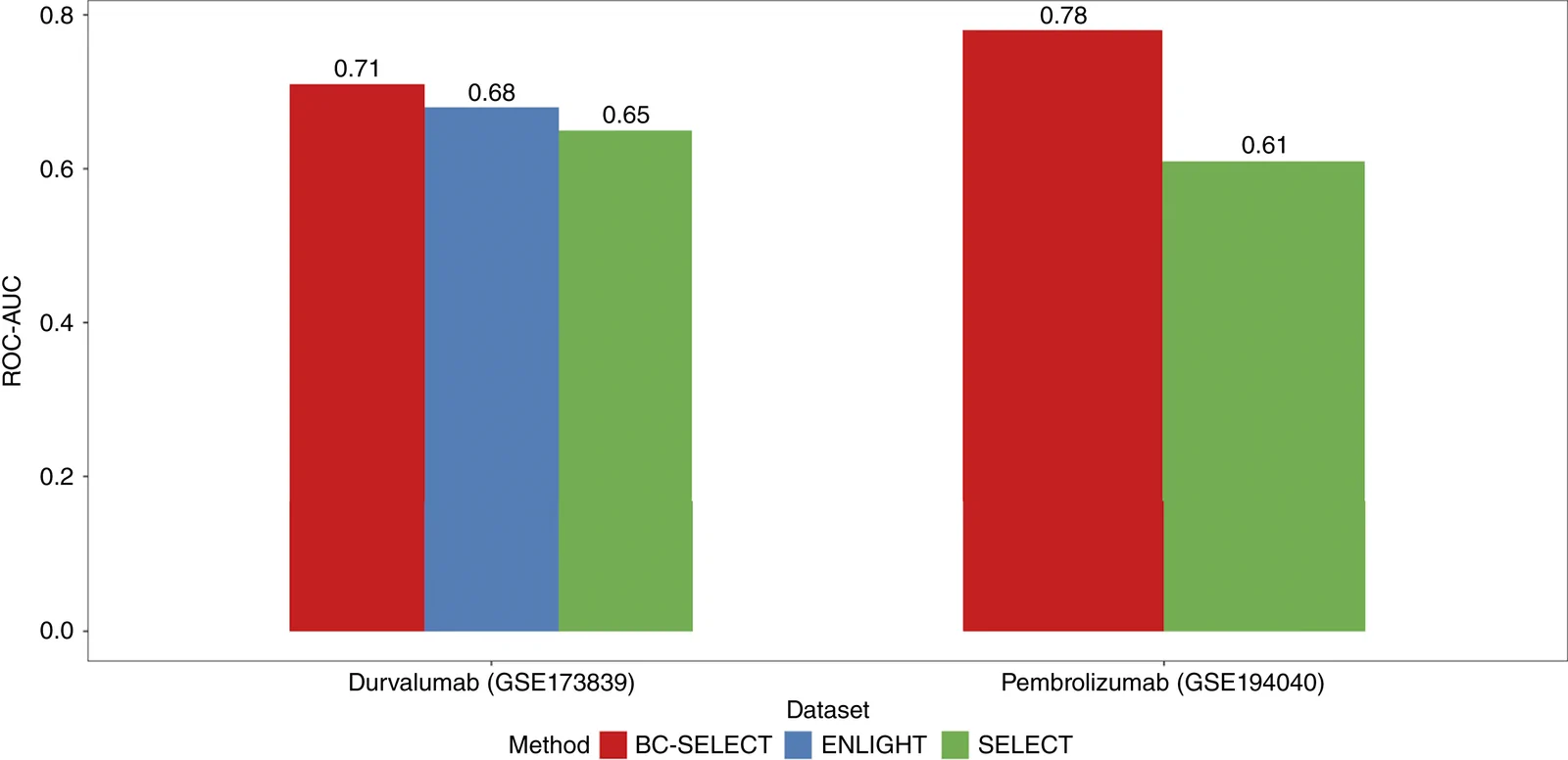
Performance comparisons between BC-SELECT and other algorithms in the context of immunotherapy. Red, BC-SELECT; blue, ENLIGHT; green, SELECT. *X*-axis shows the specific clinical trial, and *Y*-axis shows the ROC-AUC. SELECT was compared with BC-SELECT with both trials. ENLIGHT was compared using durvalumab as this was a trial used by the developers for their validation. BC-SELECT outperforms SELECT/ENLIGHT in ROC-AUC values for both validation immunotherapy trials.

BC-SELECT achieved higher ROC-AUCs than SELECT and/or ENLIGHT across two immunotherapy trials (Fig. 4). We were able to compare ENLIGHT’s performance with durvalumab in breast cancer, not for pembrolizumab, as precomputed data by ENLIGHT were available.

### BC-SELECT shows comparable specificity across subtypes

We assessed the specificity of the algorithm across breast cancer subtypes as possible. Among 550 patients with individual-level subtype data (347 with TNBC and 203 with HER2+), specificity was comparable, with 0.66 for TNBC and 0.64 for HER2+. Three trials (including both immunotherapy trials) recruited patients with HR+ disease. Specificity of BC-SELECT in HR+ disease (172 patients) was 0.76.

### Relationship between immunotherapy and PARP inhibitor BC-SELECT scores in putative BRCA-deficient tumors

**Figure 5.**
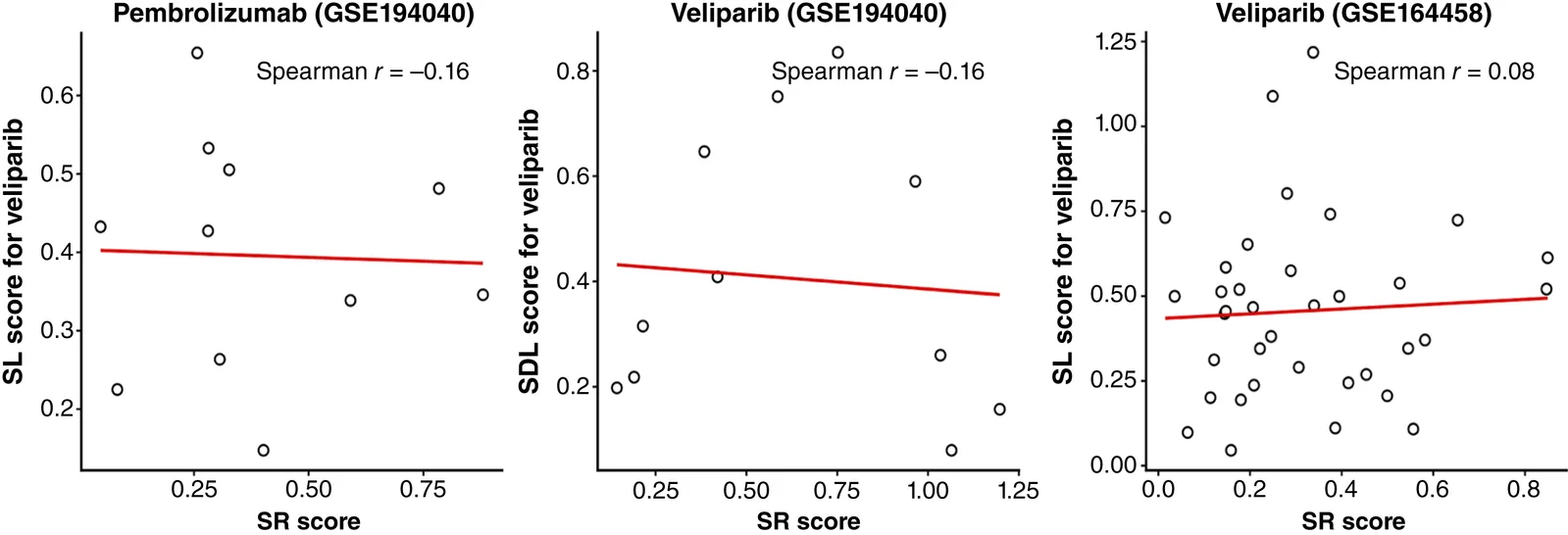
Association between immunotherapy SR scores and veliparib-related SL/SDL scores across three breast cancer cohorts. To investigate potential overlap between patients predicted to benefit from immunotherapy vs. PARP inhibitor, we calculated veliparib-related SL scores in an immunotherapy-treated cohort (GSE194040 pembrolizumab) and immunotherapy-related SR scores in PARP inhibitor–treated cohorts (GSE194040 veliparib and GSE164458 veliparib). Red lines represent fitted linear regression trends. Spearman correlation coefficients are displayed in each panel.

To explore whether BC-SELECT scores could help distinguish between immunotherapy and PARP inhibitor benefit in tumors with putative BRCA deficiency, we compared immunotherapy-related SR scores with veliparib-related SL/SDL scores among samples with low *BRCA1/2* expression (bottom 15%) across three independent breast cancer cohorts (Fig. 5). In the GSE194040 (20) pembrolizumab cohort, higher pembrolizumab SR scores were associated with lower veliparib-related SL scores. A similar inverse relationship was observed between SR scores and veliparib-related SDL scores in the GSE194040 (20) veliparib cohort. In contrast, little association was observed in the GSE164458 (59) veliparib cohort.

Because higher SR scores and higher SL/SDL scores are each associated with increased probability of pCR to their respective therapies, the observed inverse relationships suggest that patients predicted to benefit from immunotherapy may not necessarily be those predicted to derive the greatest benefit from PARP inhibition. Although exploratory, these findings raise the possibility that BC-SELECT scores could help prioritize treatment strategies within BRCA-deficient tumors, a setting in which clear biomarkers for selecting between immunotherapy and PARP inhibitors remain limited.

## Discussion

BC-SELECT is a computational algorithm that predicts complete responders to PARP inhibitors and immunotherapy in patients with nonmetastatic breast cancer. This algorithm adds to predecessors using SL and gene expression by predicting PARP inhibitor therapy response and deepening applicability in breast cancer for multiple (potentially competing) treatment options.

One hypothesis-generating application of BC-SELECT is prioritizing targeted therapy/immunotherapy where multiple options are available, as with nonmetastatic TNBC. Germline *BRCA1/2* breast cancer is the natural application for BC-SELECT. Current standard of care recommends managing these patients uniformly. We provide initial exploratory data in our results that suggest heterogeneity in *BRCA1/2-*associated breast cancers, in which not all patients will respond similarly, and that positive response to PARP inhibitors may not necessarily correlate with positive response to immunotherapy. Although preclinical data suggested that this combination could be synergistic, in practice, when tested in the metastatic setting, response was comparable with PARP inhibitor therapy and side effects were comparable with those of immunotherapy. In phase IIb JAVELIN, which used talazoparib and avelumab, the objective response rate (ORR) was 30.3% (95% CI, 22.2%–39.3%). In the MEDIOLA (15) study, which used olaparib and durvalumab, the ORR at week 12 was 63.3% (48.9%–80.1%), with a median duration of 9.2 months (95% CI, 5.5–13.1). The primary endpoint was not met in KEYLYNK, which used olaparib and pembrolizumab, with 19.3% of participants (comparable with immunotherapy) sustaining immune-mediated adverse events (61). Although, of course, difficult to directly compare, the ORR for olaparib in OlympiAD was 59.9% (95% CI, 52–67.4), with a median duration of 6.4 months [interquartile range: 2.8–9.7 months (27)].

We sought to identify patients who may not be as responsive to approved therapies and, if eligible for multiple options, may benefit from sequential ordering of treatments in order of most likely to treat their cancer. Sequential prioritization and benefit were recently described by Safonov and colleagues (62), wherein patients whose breast cancers have concurrent germline BRCA2 and somatic RB1 loss were shown to likely benefit from prioritization of PARP inhibitors over cyclin-dependent kinase (CDK) 4/6 inhibitors to avoid therapy resistance. BC-SELECT would be applied to gene expression data from tissue at the time of diagnosis and could adjust treatment with immunotherapy and PARP inhibitors as follows:

- More likely to achieve pCR with immunotherapy: Completing immunotherapy would be emphasized over other adjuvant medications.
- Less likely to achieve pCR with a PARP inhibitor: PARP therapy would be deferred until after immunotherapy.
- More likely to achieve pCR with a PARP inhibitor: potential population for future trials of neoadjuvant PARP inhibitors.

We are actively establishing collaborations to validate BC-SELECT prospectively using treatment-naïve data from patients with hereditary breast cancers in this context.

Another hypothesis-generating application would be for potential treatments outside of current clinical guidelines. We observed stronger performance in BC-SELECT in HR+ disease relative to TNBC or HER2+. This was in the context of three validation trials and will need follow-up analysis. However, this notably occurred with immunotherapy, to which HR+ disease has historically been resistant when using existing biomarker selection. Additionally, gene expression tools in HR+ predict chemotherapy benefit, but equivalent tools do not exist to prioritize possible adjuvant targeted therapies.

Of the partner genes for PARP inhibitors and immunotherapy that have been researched in the context of cancer treatment, data supporting their use in context with these treatments are available. Five of the 10 partner genes [*CEP55*, *TTK*, *CCND3*, *LAG3*, and *TMEM173* (STING)] prioritized for PD-1/PD-L1 therapy have already demonstrated efficacy in studies using SL approaches (63–69). STING agonists with checkpoint inhibitors have reached the point of early-phase clinical trials based on data in TNBC (70). Of the partner genes for veliparib and olaparib with known targeting agents, *SMARC4A* and *EZH2* both have well-demonstrated synthetic lethal effects with PARP inhibition (71, 72). Data are pending on how well PARP inhibitors may work with paired/in the setting of *ESR1* targeting therapies. Many of the partner genes, however, have not been investigated at all as related to SL or gene expression partners, and these should be considered reasonable candidates for further work.

Our pathway analysis of partner genes reflected known treatment principles. Trastuzumab is given most frequently with docetaxel, a microtubule inhibitor (23). Pathways for veliparib included helicase and ATP hydrolyase, both involved in DNA repair (25, 73). MHC-I downregulation increases resistance to immunotherapy (74). The gene families most represented among partner genes for PARP inhibitors were solute carriers (*SLC16A6*, *SLC27A4*, *SLC4A2*, *SLC7A14*, and *SLC7A8*) and cell-cycle inhibitors. *SLC7A11* has been implicated in ferroptosis and joint mechanism of response with PARP inhibitors, in which BRCA1/2 remain proficient (75). Among overlapping partner genes, co-targeting *FLT3*, *PLOD1*, *TRIB2*, or *TK1* have all shown increased therapeutic efficacy of PARP inhibitors or trastuzumab or both (76–80).

BC-SELECT demonstrated improvements in predicting response to immunotherapy in breast cancer relative to prior algorithms in this class. We attribute this improvement primarily to disease-specific optimization during training, which revises the gene pairs identified. SELECT (for which we have partner genes available) and ENLIGHT (for which we do not have partner genes available) predicted immunotherapy treatment based on pan-cancer datasets.

BC-SELECT’s overall performance, with predictive AUCs of 0.6 to 0.8 in unseen trials, was consistent with omics tests already used clinically in breast cancer. Polygenic risk scores achieve AUCs in the range of 0.6, and breast cancer gene expression assays achieve AUCs of approximately 0.75 (81, 82). In the context of prioritization of treatment options rather than eligibility where no clinical data exist, the AUCs of the algorithm fall within the reasonable range for potential use.

BC-SELECT was developed with the goal of eventual translation. We leveraged publicly available breast cancer–specific clinical trials and datasets that included individual-level responses to targeted therapy and immunotherapy. We added patient covariates and NanoString gene lists (to targeted therapy) to improve filtering of relevant gene partners. We used both microarray and RNA-seq data in the “parameter tuning” and validation steps to enable broad dataset use, although there were insufficient numbers to assess assay-specific performance. We added the “parameter tuning” step to improve BC-SELECT’s performance without severely limiting generalizability. Run time of BC-SELECT was within hours for large clinical trial datasets, allowing for easy scalability as a correlative in future prospective breast cancer programs/trials. Its modular design facilitates future adaptation, in which breast cancer–specific datasets with survival data can be added to the “Training: gene-pair identification” step, and clinical trials with individual-level response data can be added to the “Training: parameter tuning” step.

Given our focus on genetic interactions, other predictive approaches are complementary, but not competitive, with BC-SELECT. Other gene expression approaches, such as immune gene signatures or TNBC subtyping (a separate translational approach to clarify TNBCs; ref. 83), do not use genetic interactions. Similarly, predictors of immunotherapy response like tumor-infiltrating lymphocytes rely on pathology (84, 85).

BC-SELECT has limitations consistent with SELECT and ENLIGHT. It requires specific drug target(s) and can only be applied to targeted therapy and immunotherapy. Many chemotherapies have numerous target effects, limiting our ability to predict chemotherapy response. BC-SELECT cannot distinguish between therapies with the same targets. BC-SELECT will not be effective if a drug’s mechanism of action in a given cancer type does not involve SL. For example, in breast cancer, trastuzumab works most effectively in cells expressing HER2. This may explain why we did not observe strong performance with trastuzumab in this study, compared with SELECT and ENLIGHT, which trained across cancer types.

BC-SELECT was limited by data availability. We sought datasets in breast cancer with individual-level gene expression, treatment type, response, and clinical outcomes. We observed that studies with less than 25 patients in total showed greater variability and reduced discriminatory power in distinguishing pCR from non-pCR. BC-SELECT was positioned to adapt as this data landscape evolves. For TNBC and HER2+, pCR as outcome was overrepresented, whereas longer-term outcomes comparable with those of HR+ gene expression tools, such as recurrence-free/progression-free/disease-free survival, were not accessible or available. We could not access publicly available individual-level treatment response data on CDK4/6 inhibitors. PIK3CA inhibitors and ESR1 inhibitors are not standard of care in the nonmetastatic breast cancer setting. Metastatic breast cancer is the other next priority for BC-SELECT.

We present BC-SELECT, a computational algorithm to predict the extent of response to PARP inhibitors and immunotherapy in patients with nonmetastatic breast cancer. Given the increased success rates of oncology trials studying SL outside of PARP inhibitors (86), applications that use genetic interactions such as SL to select treatments merit additional consideration. BC-SELECT demonstrates predictive performance for multiple approved therapeutic options, enabling possible identification of potential complete responders and decision support for sequential therapy order that will require further clinical validation.

## Supplementary Material

Supplementary Methods Supplementary Methods

Supplementary Figure 1 Supplementary Figure 1

Supplementary Figure 2 Supplementary Figure 2

Supplementary Table 1 Supplementary Table 1

Supplementary Table 2 Supplementary Table 2

Supplementary Table 3 Supplementary Table 3

## Data Availability

All transcriptomic and clinical information analyzed in this study is publicly available as individually described in this article and/or referenced in Supplementary Table S1 (datasets). Additional details are provided in “Key resource identifiers.” Any additional data that may be required to reanalyze the data as reported in this article are available from the corresponding author upon request.

## Code Availability

The underlying code for BC-SELECT is available on GitHub: https://github.com/yewon-kim76/BC-SELECT.
