# Supplementary material for: Transcriptome-Based Synthetic Lethality Predictions to Improve Targeted Therapy/Immunotherapy Treatment Prioritization in Nonmetastatic Breast Cancer: BC-SELECT
Source: Cancer Res Commun. 2026 Oct 13;6(10):2382–94. doi: 10.1158/2767-9764.CRC-26-0401 (PMC13659572; doi:10.1158/2767-9764.CRC-26-0401)
Supplement: Supplementary file 1 [file crc-26-0401_supplementary_figure_1_suppsf1.pdf]

Supplementary Figure 1

a.

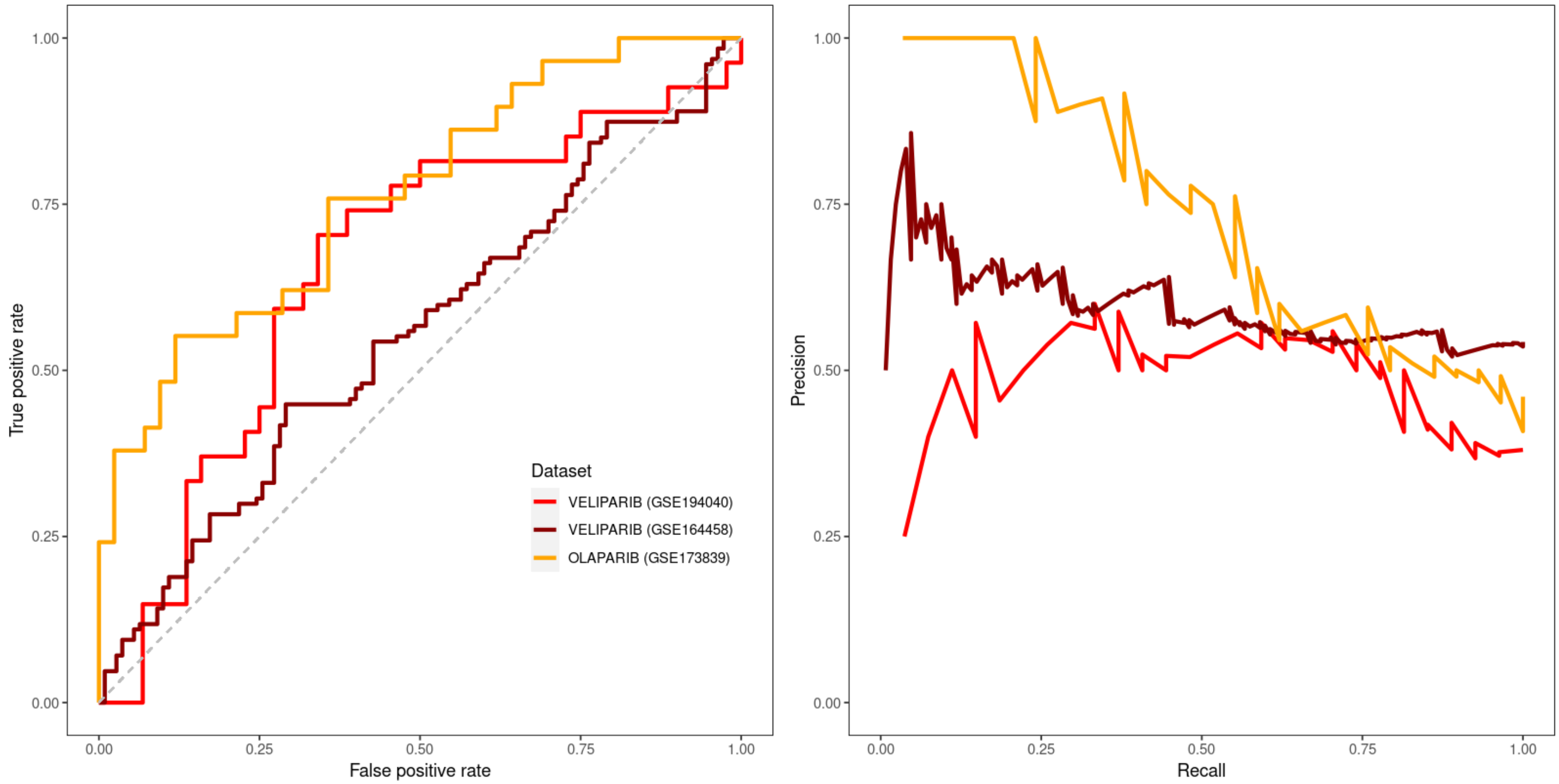

b.

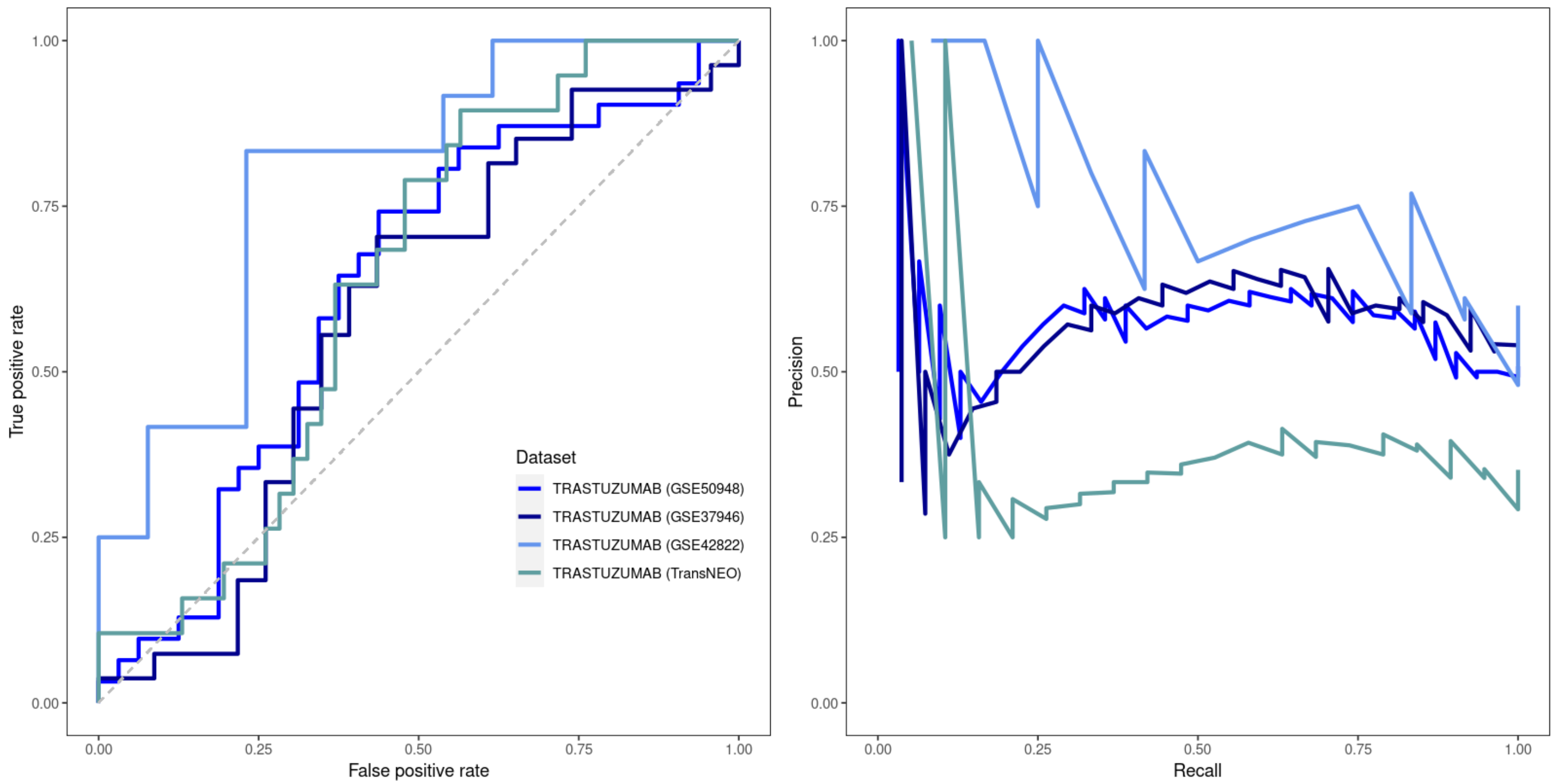

1.00
0.75
0.50
0.25
0.00
True positive rate
False positive rate
Dataset
TRASTUZUMAB (GSE50948)
TRASTUZUMAB (GSE37946)
TRASTUZUMAB (GSE42822)
TRASTUZUMAB (TransNEO)
Precision
Recall

c.

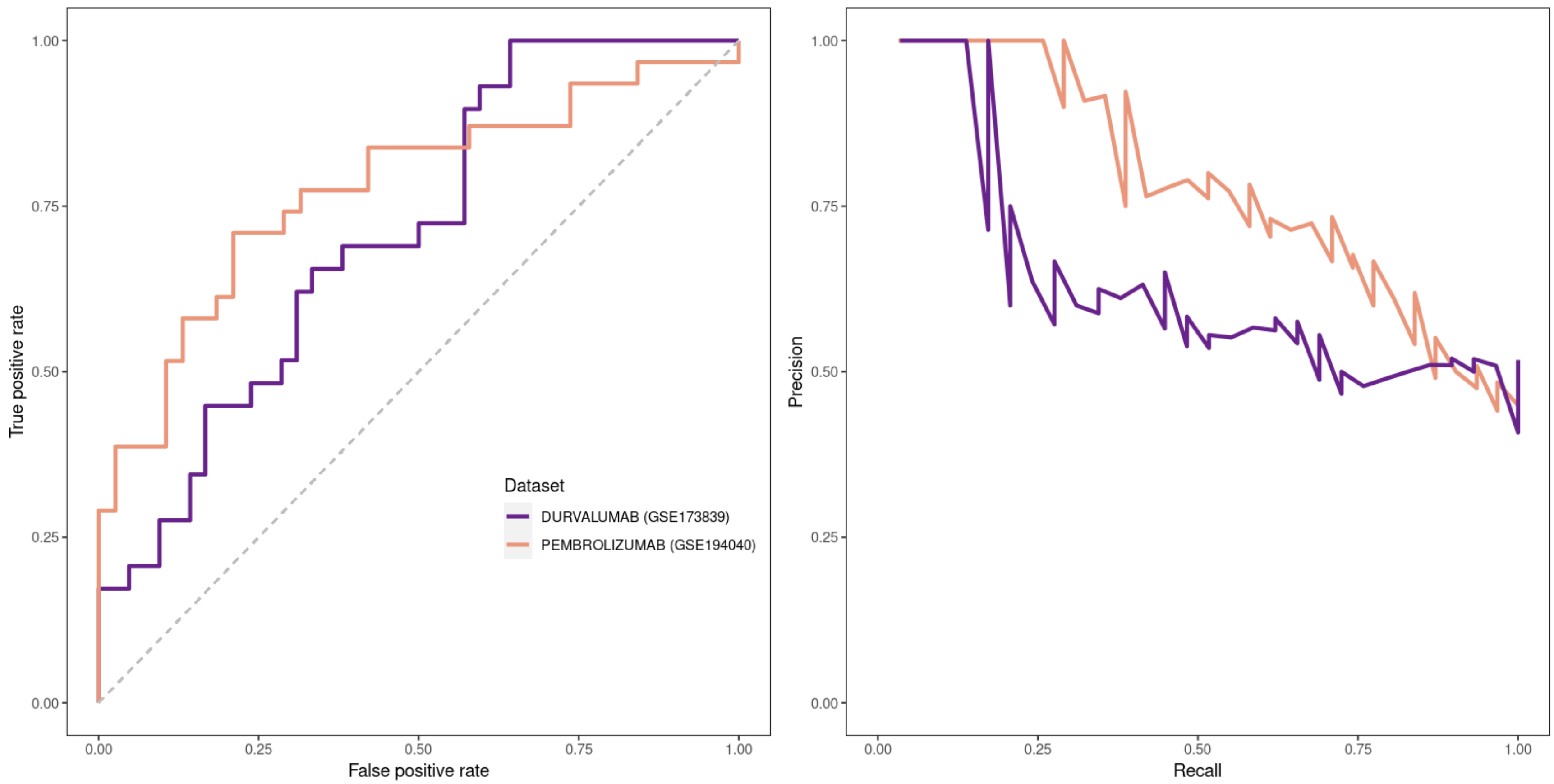

Supplementary Figure 1: ROC and precision-recall curves across therapy types. The left panel shows the false positive rate plotted against the true positive rate for the ROC curve. The right panel shows the precision recall curve. **a. PARP inhibitors.** Veliparib-based trials are in red (ROC-AUC = 0.56, 0.66) and olaparib is in orange (ROC-AUC = 0.77). **b. HER2-directed therapy.** Trastuzumab-based trials are in blue (ROC-AUC = 0.59, 0.63, 0.64 and 0.8). **c. Immunotherapy.** Durvalumab is represented in purple, and pembrolizumab is shown in pink (ROC-AUC = 0.71 and 0.78, respectively).
