# Supplementary material for: Transcriptome-Based Synthetic Lethality Predictions to Improve Targeted Therapy/Immunotherapy Treatment Prioritization in Nonmetastatic Breast Cancer: BC-SELECT
Source: Cancer Res Commun. 2026 Oct 13;6(10):2382–94. doi: 10.1158/2767-9764.CRC-26-0401 (PMC13659572; doi:10.1158/2767-9764.CRC-26-0401)
Supplement: Supplementary file 2 [file crc-26-0401_supplementary_figure_2_suppsf2.pdf]

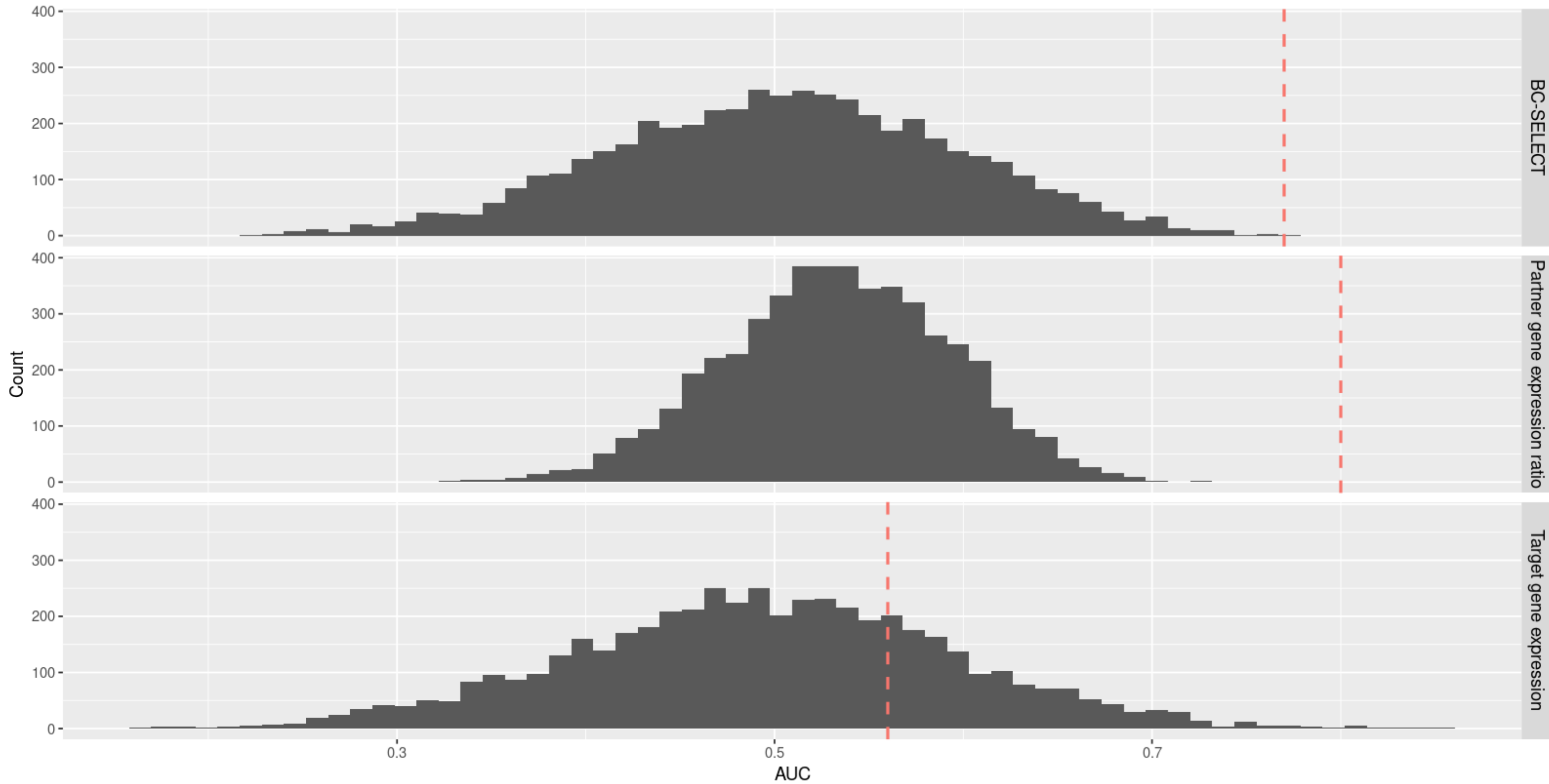

Supplementary Figure 2: Example of Monte Carlo-based “null distributions” for assessment of BC-SELECT performance of olaparib (GSE173839[75]). The figure shows the distribution of 5000 ROC-AUCs (with AUC prediction on the X-axis and number of iterations with randomly selected genes for prediction achieving that AUC on the Y-axis). The null AUC distributions are centered around 0.5, indicative of random chance classification, while their varying right-tail thickness and the central tendency reflect differences associated with the efficacy of prediction using randomly selected genes for each variable. The three red dashed lines indicate the ROC-AUC values obtained by BC-SELECT, partner gene expression ratio, and target gene expression. Empirical p-values are calculated based on the proportion of the right tail area of the red dashed line. In this example, both BC-SELECT and partner gene expression ratio exhibit smaller empirical p-values and superior performance of synthetic lethality partner genes in drug response prediction compared to chance.
