# Supplementary material for: Transcriptome-Based Synthetic Lethality Predictions to Improve Targeted Therapy/Immunotherapy Treatment Prioritization in Nonmetastatic Breast Cancer: BC-SELECT
Source: Cancer Res Commun. 2026 Oct 13;6(10):2382–94. doi: 10.1158/2767-9764.CRC-26-0401 (PMC13659572; doi:10.1158/2767-9764.CRC-26-0401)
Supplement: Supplementary file 3 [file crc-26-0401_supplementary_methods_suppsm.docx]

**Supplementary Methods

BC-SELECT Predicted Response Score**

***Partner gene expression ratio:***

The numerator is a ratio of:

Targeted therapy

- SL: Down-regulated partner genes OR
  SDL: Up-regulated partner genes over all 55 SDL gene pairs identified for a *targeted therapy*

Immunotherapy

- SR: 1 minus the ratio of down-regulated partner genes over all 10 SR gene pairs identified for *immunotherapy* (as we are measuring the likelihood of drug response)

The denominator is a ratio of:

Targeted therapy

- SL: Down-regulated genes OR

SDL: Up-regulated genes among 776 breast cancer-specific genes
for *targeted therapy*

Immunotherapy:

SR: 1 minus the ratio of down-regulated genes among 1154 immune-specific genes for *immunotherapy*

***Drug target gene expression level***

This is the gene expression level of the drug target in a given gene pair, as measured via either microarray or normalized RNA-seq. Trials or datasets were not combined to avoid batch effect correction issues. For drugs with multiple targets, we use the mean expression of all target genes in targeted therapies. For immunotherapy, we calculate the simple product of *PDCD1* and *CD274* expression.

**Monte-Carlo Simulations**

We used Monte-Carlo simulations to develop a “null distribution of AUCs” for each trial. Using 55 randomly selected gene pairs for targeted therapy and 10 randomly selected gene pairs for immunotherapy, we calculated ROC-AUC values for these randomly selected genes and iterated this process 5000 times to yield the “null distribution”. We then compared the BC-SELECT’s predictive ROC-AUC to those achieved by chance. This process produced empirical p-values, in which the top 2.5% (statistical significance threshold p=0.025), corresponds to the single tail with predicted response better than two standard deviations from the mean in the null distribution (Supplementary Figure 2).

**Changes relative to SELECT**

We adapted the original code from SELECT^1^ as follows:

- We remove copy number data to facilitate future addition of publicly available breast cancer datasets.
- We also add additional patient-specific covariates to the survival analyses in the Cox proportional hazards filtering step during the gene-pair identification step to improve clinical utility.
- For targeted therapies, we rank candidate partner genes based on their significance in the survival analysis filtering step.
- We introduce SDL as a separate concept and score, wherein a higher SDL score signifies a higher likelihood of cancer cell death and a stronger drug response.
- We have revised the calculation of the final predicted response score, including the incorporation of drug target gene expression and the partner gene expression ratio.
- We perform Monte Carlo simulations to generate empirical p-values to assess the performance of BC-SELECT relative to chance.

**Reference**

1.	Lee JS, Nair NU, Dinstag G, et al. Synthetic lethality-mediated precision oncology via the tumor transcriptome. *Cell*. 2021;184(9):2487-2502.e13. doi:10.1016/j.cell.2021.03.030
